# Extraction of impacted third molar with preventive installation of titanium miniplate: Case report

**DOI:** 10.1016/j.amsu.2019.11.014

**Published:** 2019-11-27

**Authors:** Joanna Gaba Feniar, Humberto Kawano, Gisela Cristina Vianna Camolesi, Michelle Palmieri, Sérgio De Souza Sobral, Fábio Lopes Duarte, Marcos José Barboni Maringoli, Henrique Camargo Bauer, Waldyr Antônio Jorge, Renata Matalon Negreiros

**Affiliations:** a Foundation for Scientific and Technological Development of Dentistry at University of São Paulo (FFO-USP), School of Dentistry, FOUSP, University of São Paulo, São Paulo, Brazil, Av Professor Lineu Prestes, 2227, Butantã, CEP:05508-000, São Paulo, SP, Brazil; b Department of Stomatology, School of Dentistry, University of São Paulo, São Paulo, Brazil; c Department of Biophotonics Applied to Health and Sciences, University Nove de Julho, UNINOVE, São Paulo, Brazil; d Integrated Clinic, Department of Estomatology, São Paulo University, São Paulo, Brazil; e University Nove de Julho, UNINOVE, São Paulo, Brazil

**Keywords:** Third molar, Tooth extraction, Mandibular fractures, Plate systems

## Abstract

**Introduction:**

Even though is rarely, mandibular fracture after the extraction of third molars can occur in almost 1% of the procedures. Gender, age, position of third molar, tooth volume, bone fragility, systemic disorders, associated lesions, and degree of mandibular atrophy are factors that contribute to increase the incidence of fracture. The installation of the titanium miniplate during exodontia is an important tool to prevent the fracture.

**Presentation of case:**

The objective of this study is to present a clinical case of extraction of inferior impacted third molar, in atrophic mandible, with posterior installation of titanium miniplate, to prevent mandibular fracture.

**Discussion and conclusion:**

preventive installation of titanium miniplate was effective and indeed prevented the mandibular fracture. No trans-operative or immediate post-operative complications were observed. Post-operative follow-up was of three years, with no complications, showing the success of the procedure.

## Highlights

• This study will instruct surgeons on third molar surgery in atrophic mandible.
• It will allow surgeons to avoid mandibular fracture, by installing titanium plate.
• It will ensure the procedure is safe and well conducted for the patients.

## 1 Introduction

Inferior third molar extraction is the most performed procedure by oral maxillofacial surgeons, however it may be associated with several complications: alveolitis, hemorrhage, infection, paresthesia, trismus and rarely mandibular fracture [1,2].

Mandibular fracture is a uncommon complication, with low incidence after third molar extraction (<1%). It is more frequently described in the literature in male patients, above 40 years old, presenting systemic disorders (hyperparathyroidism and Pajet disease), with large tooth volume and in impacted position (class II and III, type B and C, according to Pell and Gregory classification – 1933). It is usually associated with atrophic mandible with impacted lower third molar (associated or not with bone cyst or tumors) [3].

In relation to the procedures involved, it is associated with poor surgical planning of tooth extraction and inadequate instrumentation. It also may occur, when excessive manual force is applied during the extraction procedure. In those cases, the extraction's region leads to an area of bone fragility. This fact, in addition to a combination of forces from the masticatory muscles (over a possible fragile mandible) may increase the incidence of fracture [[1], [2], [3]].

To prevent mandibular fracture, the rigid fixation system with miniplate may be an important alternative measure to be used [3,4].

The objective of this study is to present a clinical case of extraction of inferior impacted third molar, in atrophic mandible, with posterior installation of titanium miniplate, to prevent mandibular fracture, illustrated with a clinical case. This work has been reported according to the SCARE criteria [5].

## 2 Presentation of case

The study was approved by the Research Ethics Committee of University of Sao Paulo number #2865397. Informed consent was obtained from the participant included in this study.

**Fig. 1:**
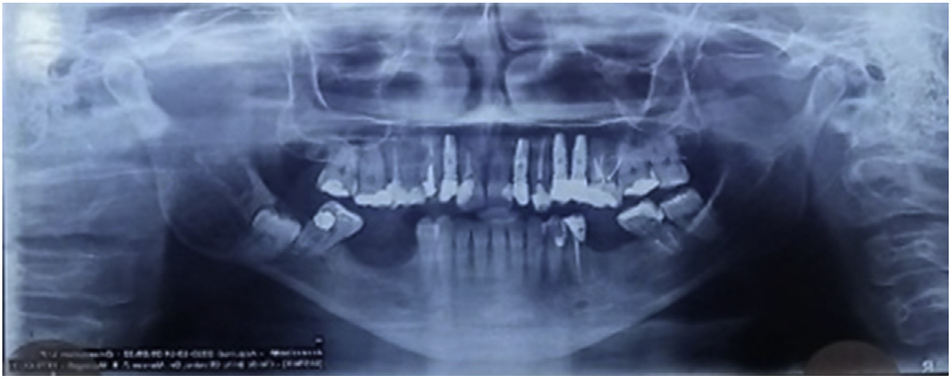
Pre operative radiography.

**Fig. 2:**
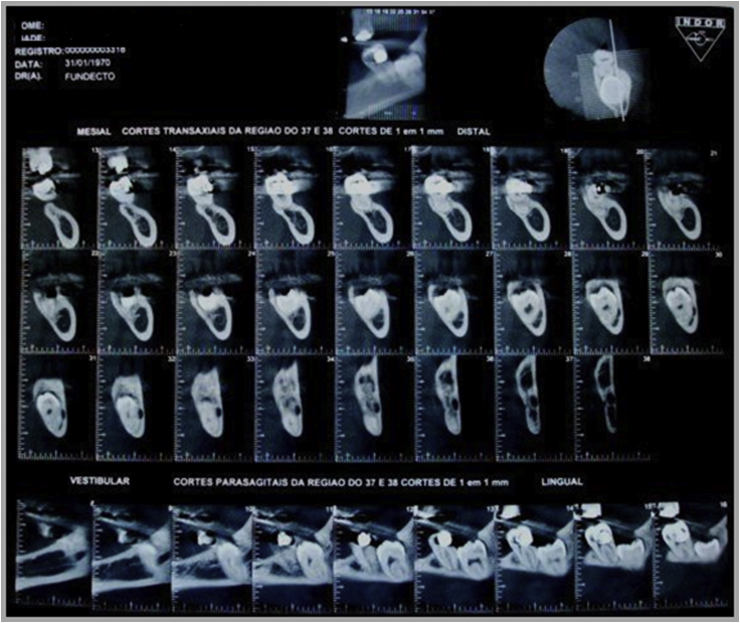
Pre operative CT.

A 45-year-old female patient, caucasian, with no previous systemic disorders, was evaluated in the Specialization of Oral and Maxillofacial Surgery at Foundation for Scientific and Technological Development of Dentistry at University of São Paulo (FFO-USP). On radiographic examination, the lower third molar was impacted in class III, type C (according to Pell and Gregory classification). Tooth roots were located in the basilar region of mandible and bone thinness in the basal mandible region was observed (Fig. 1). The pre operative computer tomography confirmed the bone thinness at the buccal side of the third molar area (Fig. 2). The surgical plan was an exodontia of the lower third molar and installation of a titanium miniplate in order to prevent the late mandible fracture.

The surgical procedure steps for tooth extraction, were performed in the following order: local anesthesia with Mepivacaine 2% with adrenaline (Nova DFL, Rio de Janeiro - Brazil) Winter's incision, mucoperiosteal flap divulsion, minimal vestibular and distal ostectomy, section of the tooth vertically separating the roots, avulsion of the mesial tooth portion and in the sequence the distal tooth portion avoiding excessive forces, using piezo electrical hand piece, curettage of the alveolus and irrigation with saline solution.

**Fig. 3:**
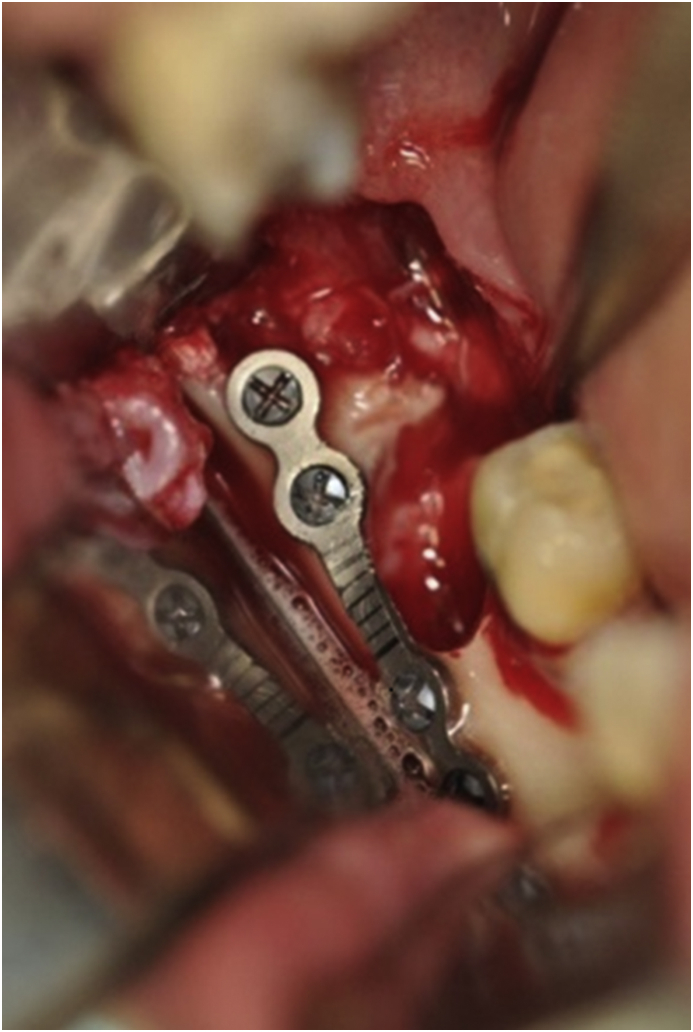
Miniplate 2.0 mm installed at mandibular angle.

After the tooth extraction the installation of the titanium miniplate according to the Champy's technique was performed. The titanium miniplateplate available at the FFO-USP department was a 4-holes bridge miniplate 2.0 screw system (2 mm × 5 mm x 20 mm), with monocortical screws of 6 mm (Osteomed, Rio Claro - Brazil) was installed by intraoral access. . The miniplate was adapted on the external oblique line, to promote reinforcement in the tension's bone zone and prevent late mandibular fracture (Fig. 3). After installation of the mini-plate, a extra irrigation with saline solution was performed and finally sutured using 3-0 monofilament silk (Ethicon Johnson & Johnson, São Paulo - Brazil). No trans-operatory or immediate postoperative complications were observed.

**Fig. 4:**
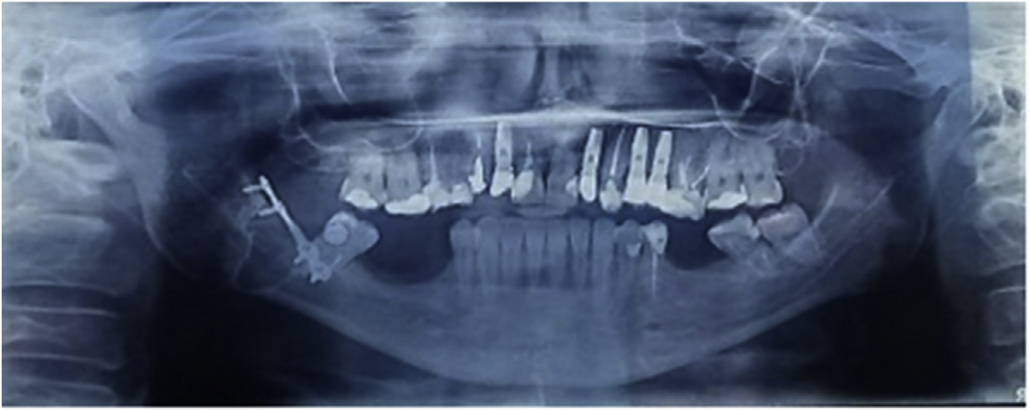
Post operative Radiography (7 days).

Postoperative recommendations were explained to the patient, including strict instruction to maintain a soft diet extended for a period of 4 weeks. Patient was medicated with antibiotics, for a period of 7 days, and anti-inflamatory and analgesic for 5 days, Sutures were removed after 7 days, reduction in the swelling and no sign of infection was observed. The post operative radiography showed that the miniplate was still installed in the correct position (Fig. 4). On the first month the follow up visits were conducted weekly, showing total reduction of the swelling and no sign infection or paresthesia.

**Fig. 5:**
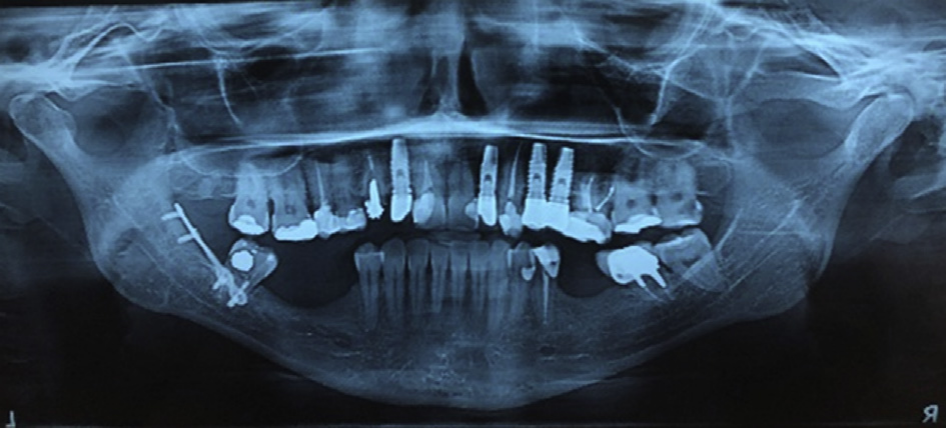
Radiography of 3 years follow up.

On the next 6 months, the follow up visits were conducted monthly, and after that, once a year. At 1 year follow up, patient did not complain about pain, paresthesia or infection. On the last follow up visit, conducted after 3 years of surgery, it was observed on the panoramic radiograph that the miniplate was still installed in the correct position and there was complete bone formation in the region of the tooth's extraction (Fig. 5).

## 3 Discussion

The incidence of mandibular fracture after the extraction of third molar (late fracture) is uncommon (<1%), however it can happened in a few occasions [2,3,6]. In the literature, most authors report the occurrence of late fracture, between the second and forth week after exodontias, during chewing, specially at meal times [6]. It is was also reported that patients from the male gender and above 40 years of age, present a higher incidence of late mandibular fracture [[1], [2], [3],^7^].

Usually, the fracture is associated with atrophic mandible, with impacted lower third molar, with large tooth's volume (associated or not with bone cyst or tumors) [3,7], poor surgical planning, inadequate technique, inadequate instrumentation and the use of excessive manual force during the extraction procedure.

The presence of the third molar at the angle of the mandible directly influences the mandibular resistance. In those cases, the extraction's region leads to an area of bone fragility. In addition, a combination of forces from the masticatory muscles (masseter, medial pterygoid and digastrics), over a possible fragile mandible, is transmitted to the mandible through the teeth, what may increase the incidence of fracture [[1], [2], [3],^8^,^9^]. In face of the potential incidences of late mandibular fractures, patients must be informed clearly to maintain soft diet for at least 4 weeks [8]. In our case, extraction was indicated in a female patient, however she was 45 years old and the third molar was impacted in class III, type C (according to Pell and Gregory classification), representing a surgical procedure that deserves attention.

Additional causes, other than the chewing phenomenon may lead to late mandibular fractures, such as systemic disorders that causes bone's fragility (osteoporosis, hyperparathyroidism, rheumatism, osteogenesis imperfecta and Pajet disease) and local trauma with impact on the surgical site [1,2,7,9].

During the extraction, it is worth mentioning that it is always preferable to perform as many tooth sections as necessary, minimizing ostectomy and, consequently, reducing the fragility of the mandible and the incidence of fracture [3,10,11]. It is described in the literature that the use of piezo electrical handpiece for osteotomy is indicated to reduce excessive forces during tooth avulsion and reduce manipulation of the neural structures, avoiding paresthesia [12]. In our report we performed a minimal ostectomy, tooth section and the piezo electrical handpiece was used. The procedure was successful and patient did not complain of paresthesia on the post operative follow up.

In order to prevent the occurrence of a mandibular angle fracture, it may be recommended the fixation of miniplate pre or post extraction to reduce the incidence of fracture [[3], [4], [5],^9^,^11^,^13^]. In the literature, there are two philosophies of miniplate fixation: Champy and AO/ASIF (ArbeitsgemeinschaftfürOsteosynthesefragen/Association for the Study of Internal Fixation). The Champy technique consists of the use of only one minplate in the region of oblique external line with intraoral access and monocortical screws [14]. The AO/ASIF technique uses two miniplates, one in the tension zone with monocortical screws (alveolar process) and the other in the compression zone with bicortical screws (mandibular base), through trans buccal or external accesses [15].

Regarding the type of plaque used to prevent a possible mandibular fracture, the use of miniplate and titanium screws of system 2.0 is satisfactory. In relation to surgical access to the mandible's posterior body and to the mandibular branch, the most recommended is the submandibular access or Risdon. This access may present to the surgeon a broad view of the operative field, in a relatively safe technique, with low index of contamination considering the microbiota of the buccal environment. However, has the disadvantage of propitiating the external scar and the incidence of facial nerve injury [4,11,15]. In this study, the surgical access was intraoral, allowing the procedure of tooth extraction and the installation of the titanium mini plate, through de same access. This choice was due to aesthetic issues, one of the concerns of the patient and prevention of facial nerve damage.

The installation of the titanium mini plate was performed after the exodontia, in order to avoid interference in direct visualization of the site and provide proper surgical maneuvers during the extraction procedure [4,9,15]. Furthermore, it is described in the literature, that the higher incidence of mandibular fracture associated to third molars extractions, occur between 15 and 30 days in the postoperative period [3], justifying our decision in relation to the moment of miniplate's installation.

## 4 Conclusion

Although rare, mandibular fracture may occur after the extraction of lower third molars, especially when it is associated with atrophic mandible with impacted tooth, leading to an area of bone fragility.

The lower third molar extraction with posterior miniplate installation, according to Champy technique, was an alternative measure to prevent mandibular fracture, that was effective with satisfactory results.

## Ethical approval

All procedures performed in this study involving human participants were in accordance with the ethical standards of the institutional and/or national research committee and with the 1964 Helsinki declaration and its later amendments or comparable ethical standards. The study was approved by the Research Ethics Committee of University of Sao Paulo number #2865397. **Informed Consent** Informed consent was obtained from the participant included in this study.The identity of the patient will not be disclosed. Individual data of patient will be protected in order to protect her confidentiality.

## Sources of funding

This study was carried out with funding from the researchers themselves.

## Author contribution

**JOANNA GABA FENIAR** - has made substantial contributions to conception and acquisition of literature; **HUMBERTO KAWANO** has done the surgical procedure; **GISELA CRISTINA VIANNA CAMOLESI** has been involved in drafting the manuscript; **MICHELLE PALMIERI** has been involved in drafting the manuscript and revising it critically for important intellectual content; **SÉRGIO SOBRAL** has made substantial contributions surgical planning and supervision **FÁBIO LOPES DUARTE** - has made substantial contributions surgical planning, has done the surgical procedure **MARCOS JOSÉ BARBONI MARINGOLI** has made substantial contributions surgical planning, has done the surgical procedure,; **WALDYR ANTÔNIO JORGE** has been involved in revising it critically for important intellectual content; **HENRIQUE CAMARGO BAUER** has been involved in drafting the manuscript and revising it critically for important intellectual content; **RENATA MATALON NEGREIROS** has been involved in drafting the manuscript and has given final approval of the version to be published.

## Registration of research studies

Researchregistry5022not applicable to case report.

## Guarantor

Renata Matalon Negreiros.

## Informed consent

All procedures performed in this study involving participants were in accordance with the ethical standards of the institutional and/or national research committee and with the 1964 Helsinki declaration and its later amendments or comparable ethical standards. This study was approved according to protocol number 2865397by the Research Ethics Committee of University of São Paulo. Written informed consent was obtained from the patient for publication of this case report and accompanying images. A copy of the written consent is available for review by the Editor-in-Chief of this journal request.

## Provenance and peer review

Not commissioned externally peer reviewed.

## Declaration of competing interest

All authors declare that they have no conflict of interest regarding this article.and neighter financing relationship.
